# Recovery in Borderline Personality Disorder (BPD): A Qualitative Study of Service Users' Perspectives

**DOI:** 10.1371/journal.pone.0036517

**Published:** 2012-05-17

**Authors:** Christina Katsakou, Stamatina Marougka, Kirsten Barnicot, Mark Savill, Hayley White, Kate Lockwood, Stefan Priebe

**Affiliations:** 1 Unit for Social and Community Psychiatry, Queen Mary University of London, London, United Kingdom; 2 Together (Mental Health Charity), London, United Kingdom; 3 Therapeutic Community and Outreach, East London Foundation NHS Trust, London, United Kingdom; University of Granada, Spain

## Abstract

**Background:**

Symptom improvement in Borderline Personality Disorder (BPD) is more common than previously hypothesised. However, it remains unclear whether it reflects service users' personal goals of recovery. The present study aimed to explore what service users with BPD view as recovery.

**Methods:**

48 service users were recruited from secondary mental health services and their views on their personal goals and the meaning of recovery were explored in in-depth semi-structured interviews. The study drew on grounded theory and thematic analysis.

**Results:**

Service users believed that recovery involved developing self-acceptance and self-confidence, gaining control over emotions, improving relationships, employment, and making progress in symptoms like suicidality and self-harming. They felt that psychotherapies for BPD often had an extreme focus on specific areas, like self-harming or relationships, and that some of their goals were neglected. Although full recovery was seen as a distant goal, interviewees felt that they could learn how to deal with their problems in more effective ways and make meaningful progress in their lives.

**Conclusions:**

Specialist therapies for BPD explicitly address some of the recovery goals that are important to service users, whereas other goals are only indirectly or poorly addressed. Professionals might need to work with service users towards devising comprehensive individualised case formulations, including all treatment targets that are important to service users, their priorities, and long-term plans on how their targets might be met and which services might be involved.

## Introduction

Borderline personality disorder (BPD) has long been a burden for those suffering from the condition and a challenge for clinicians. The prevalence of the disorder is between 1% and 5.9% in the general population [1]–[5]. Individuals with BPD experience great difficulties in regulating their emotions, unstable relationship patterns, mood swings, feelings of emptiness and chaotic lifestyles. Suicide attempts and/or self-harming are common in 69–80% and completed suicide occurs in up to 10% of those diagnosed [6]–[8]. Service users with BPD consume significant therapeutic resources [9] and professionals treating them often feel overwhelmed and distressed [10]–[11]. Self-harming behaviour is one of the main reasons for psychiatric hospitalisation and other costly interventions [10].

BPD was considered by many to be chronic and unresponsive to treatment [12]. However, recent evidence indicates that the severity of BPD symptoms among those receiving treatment in mental health services decreases dramatically over time. Studies from the USA and UK indicate that under half of patients initially meeting criteria for BPD still do so 6 years later [13] whilst after 10 years this drops to 26% [14]. Evidence from randomised controlled trials shows that several specialist psychotherapies for BPD are effective in reducing symptoms [13], [15]–[26]. These have included Dialectical Behavioural Therapy (DBT) and Mentalization-based Therapy (MBT), which can lead to reduction in suicide attempts and self-harming, and less use of crisis services [15]–[20].

Although such clinical improvements are an important achievement and an obvious target for services, it remains unclear whether they reflect service users' perceptions of personal recovery and desired outcomes. It has been observed that clinical improvement or risk reduction, traditionally assessed in mental health research, do not always coincide with patients' personal evaluations of recovery and meaningful progress in their lives [27]–[28]. Personal recovery is often seen as a way of *‘living a satisfying, hopeful, and contributing life even with the limitations caused by the illness’* [29].

Furthermore, recovery might be interpreted differently by different groups of service users. Although qualitative studies have explored the meaning of recovery for users of general psychiatric services with a diagnosis of Axis 1 disorders (depression, schizophrenia, bipolar disorder etc.) [30], little is known on what service users with BPD see as recovery. Recent evidence indicates that service users of specialist services might have recovery goals that are more closely linked to their specific diagnosis and needs, rather than to goals identified by users of generic services [30].

The present study explored what people with a diagnosis of BPD view as recovery. Determining important personal goals and aspirations for service users might facilitate the further development of existing specialist psychotherapies and the delivery of routine care for this challenging group. It might also ensure that treatments prioritise targets that are relevant to service users, and therefore help them maintain their motivation to make meaningful changes in their lives.

## Methods

### Design

An exploratory, qualitative, interview-based study, assessing patients' perspectives of recovery in BPD was conducted. The study design drew on Grounded Theory and thematic analysis. Grounded theory is a method aiming to inductively build a theoretical explanation of a social phenomenon based on the study data [31]–[33]. Thematic analysis is used to identify a limited number of themes that adequately reflect the data, by comparing and refining emerging topics [34].

The core research team included researchers with academic and clinical backgrounds and service users. More specifically, CK is an academic researcher and a DBT psychotherapist; SM works clinically with forensic patients with personality disorders; KB and MS are academic researchers working with service users with a BPD diagnosis; SP has a long clinical and research experience as a psychologist and psychiatrist; HW is a service user with a diagnosis of BPD who has received MBT; KL is a consultant psychiatrist and psychodynamic psychotherapist working in an MBT-informed setting. The team met regularly to discuss the study design, implementation, and data analysis.

Service-users were also involved in various stages of the study (design, data analysis and interpretation) to ensure that their perspectives were reflected in the interpretation of the data. Their specific contributions will be described in detail in the relevant parts of the methods.

Written informed consent for participation in the study was obtained by all interviewees. The study design was approved by the East London National Health Service (NHS) Research Ethics Committee (ref: 09/H0704/14).

### Sample and data collection

Participants were recruited from secondary mental health services in East London, including two specialist services for BPD (a DBT team and a therapeutic community informed by the MBT approach) and generic mental health services who offer support to service users with a range of Axis 1 and Axis 2 diagnoses. The generic services included 3 community mental health teams (CMHTs) and a psychological therapies service. The psychological therapies service offers psychological therapy, including cognitive behavioural, psychodynamic and integrative interventions. The inclusion criteria for participation in the study were: age above 18 years, a diagnosis of BPD and a history of self-harming. Self-harming was defined as self-injurious behaviour, overdosing or suicide attempts that were performed with the intention to self-harm. It should be noted that participants did not have to engage in self-harming behaviour currently, but were included if they had done so at any point in their lives. A history of self-harming was used as an inclusion criterion, as we wished to have a homogenous sample and we aimed to include those who had experienced more severe BPD symptoms at some point in their lives and who are therefore likely to use services for BPD-related problems frequently. Furthermore, in our experience, service users with no self-harming behaviour might be more likely to receive Axis 1 diagnoses, such as mood, anxiety or eating disorders, and therefore psychiatric or psychological treatments not specifically developed for BPD. Those with severe learning disabilities, those who did not speak sufficient English to participate in interviews and those unable to give informed consent were excluded.

The current and archived referrals to the two specialist services were reviewed and eligible patients were identified. Professionals from three CMHTs and one psychological therapies service were also contacted and asked to inform the researchers about eligible patients. Purposive sampling was applied to ensure that the research aims were addressed and that the sample included interviewees with a wide range of characteristics. Both patients who perceived that they have recovered and those who did not were included. Similarly, participants with various clinical and demographic characteristics were selected (i.e. co-morbid diagnoses, service use, a wide range of ethnic backgrounds, age and gender). Furthermore, service users who were engaged with services and those who discontinued their treatment were interviewed. Among those who were engaged with services, we aimed to interview service users after they had used services for at least 4 months, so that they had some time to reflect on the treatments they received. New participants were recruited on the basis of their potential similarities or discrepancies from the already participating sample. The sampling of new participants stopped when saturation of the emerging themes was reached [31]–[33].

Once patients meeting the inclusion criteria were selected, they were contacted through their key-worker and introduced to a researcher. The researcher explained the study and asked for their informed consent to take part. If consent was given, self-reported socio-demographic and clinical characteristics were collected (see table 1) and the qualitative interviews were conducted. The clinical characteristics included diagnosis as documented in the service users' files. Both specialist services used SCID-II to assess Axis 2 diagnoses, whereas in generic services all diagnoses were commonly given by psychiatrists in clinical interviews (without the use of psychometric instruments).

**Table 1:** Participants' socio-demographic and clinical characteristics.

	Total sampleN (%)
**Gender**	
Female	39 (81)
Male	9 (19)
**Age**	
mean (SD)	36.5 (10.38)
**Ethnicity**	
White	33 (69)
Black	5 (10)
Asian	10 (21)
**Employment**	
Unemployed	37 (77)
Voluntary work	3 (6)
Employed	8 (17)
**Accommodation**	
Independent accommodation	48 (100)
**Partnership**	
Living alone	28 (58)
Living with partner/family	20 (42)
**Co-morbid Diagnoses**	
Avoidant PD	25 (52)
Dependent PD	10 (21)
Obsessive compulsive D	20 (42)
Paranoid PD	22 (46)
Schizotypal PD	7 (15)
Schizoid PD	6 (13)
Histrionic PD	1 (2)
Narcissistic PD	6 (13)
Antisocial PD	8 (17)
Depression/dysthymia	21 (44)
Bipolar disorder	4 (8)
Schizoaffective disorder	4 (8)
Eating disorder	6 (13)
Anxiety disorder (PTSD, OCD, phobia)	8 (17)
Alcohol/drugs abuse	8 (17)
**Treatment**	
DBT	23 (48)
MBT	8 (17)
Other psychological therapy	6 (13)
Generic services	11 (23)
**Treatment completion** *	
Completed	28 (76)
**Received counselling/psychotherapy at least once in the past**	44 (92)
**Years in mental health services**	
0–5 years	28 (58)
6–10 years	16 (33)
11–15 years	4 (9)

* only applicable to those receiving psychological therapy.

The sampling and data collection process was discussed in a meeting with CK and 4 service users who had used both generic and specialist services. Their feedback informed final decisions in these areas.

### Interviews and topic guide

In depth semi-structured interviews were conducted. A topic guide for the interviews was developed in a meeting between CK and two service users who had used generic and specialist services. Participants were asked to describe what they perceived as recovery, their goals and aspirations, their journey towards recovery and their reflections on their progress and achievements.

The above-mentioned core topics were covered in all interviews. Participants were also encouraged to discuss related topics that they judged significant. The interview style was flexible, guided by neutral and open questions. The interviews lasted between 30 and 120 minutes. Four researchers recruited and interviewed study participants. All interviews were recorded using a digital recorder and transcribed by a professional transcriber.

### Data analysis

Data gathering and analysis were interdependent and were carried out one after the other repeatedly, until saturation on the meaning of recovery from BPD was reached. This was a dynamic process, consisting of moving from data collection, through initial analysis, to theoretical hypothesis generation that informed new rounds of sampling and data collection. Thematic interview coding and constant comparisons to identify similarities and differences between emerging themes and sampled cases guided the researchers into more abstract understandings of the themes, leading to their conceptual clarification and to the development of more holistic interpretations [31]–[34].

The process of the construction of a coding frame to capture the various emerging themes was inductive and based on the interview data. The coding frame was developed by four researchers and two service users. Following this, three researchers coded 30 interviews together to discuss practical issues around coding and refine the coding frame, a technique known as multiple coding [35]. CK then coded all interview transcripts using the MAXqda software (version 2) for qualitative data analysis.

Once the research team had agreed on the core themes emerging from the data, the data analysis was discussed in a meeting between CK and 4 service users. Their feedback helped the research team to further refine their understanding and interpretation of the data.

## Results

Out of the 54 eligible service users that were invited to participate in the study four refused to do so and two agreed to take part but did not attend their scheduled appointment for an interview. The remaining 48 (89%) were interviewed. Participants' socio-demographic and clinical characteristics are presented in table 1.

Various topics linked to interviewees' perceptions of recovery were identified and will be presented below. Firstly, participants discussed their personal goals and/or achievements during recovery and how these relate to service targets. Secondly, they reflected on their current stage of recovery. Lastly, they reported some concerns regarding the use of the word ‘recovery’. The frequency of these themes among study participants is presented in table 2.

**Table 2:** Participants' perspectives of recovery in Borderline Personality Disorder (BPD).

	Total sampleN (%)
**Personal goals and/or achievements during recovery**	
Accepting self and building self-confidence	32 (67)
Taking control of emotions, mood and negative thinking	40 (83)
Improving relationships	28 (58)
Practical achievements and employment	24 (50)
Reducing suicidality, self-harming and other symptoms	37 (77)
**Total number of goals/achievements**	
0	3 (6)
1–3	15 (31)
4–5	30 (63)
**Tension in balancing personal goals of recovery versus service targets**	21 (44)
**How recovered do people feel?**	
No progress	9 (19)
Recovery fluctuating	18 (38)
Able to deal with things in a better way but not (fully) recovered	40 (83)
Recovered	5 (10)
**Problems with the word ‘recovery’**	24 (50)

### A. Personal goals and/or achievements during recovery

The main areas where participants felt that they have made progress or would still like to improve are presented below. These goals/achievements were seen as inter-linked and influencing each other: improvement or deterioration in one area usually led to improvements or problems respectively in achieving other goals.

#### A1. Accepting self and building self-confidence

Participants described that they have managed or want to understand themselves more and make sense of their problems, their actions and thinking patterns, as well as the reasons underlying why they behave in certain ways. Understanding themselves and their history was seen as a step towards accepting themselves more, being less self-critical and coming to terms with who they are.

People expressed that they progressively felt more confident within themselves and less self-blaming. They wanted to let go of guilt and shame, develop a view of themselves as worthy individuals and the capacity to like themselves and feel compassion for their problems. *“I was so unhappy before. I was unsure of everything. I was always appalled with myself, there was always one thing or another that I was beating myself up about. I had nothing to offer. I was self-harming, which was my only way of dealing with things; then I was disgusted that I'd done it afterwards, but it was just horrible. I felt like I was rubbish at my job - everything. Just horrible. And now, I feel like I've got some things to offer, more confident and I'm happy. And I can talk to people about things more easily”* (participant 13).

Building on developments in self-esteem, they also wanted to feel more confident and assertive in relationships, and be able to ask for what they want. They wanted to be more competent in dealing with their problems and their lives, more independent, and gradually reduce the support they receive form mental health services. *“I feel more confident. I keep on doing something and then thinking ‘well I wouldn't have done that last year’. I'm stronger in myself, with relationships, with anything. Even when I might be talking on the phone with somebody who I don't want to talk to, like a salesman, and then stopping them in between, whereas before I would let them rant on until the end and I'd probably sign up to what they wanted me to”* (participant 4).

#### A2. Taking control of emotions, mood and negative thinking

Participants described that an important part of recovery is gaining more control over their emotions, moods and negative thoughts. They want to have more control over negative emotions such as anger, sadness, grief, emptiness, fear. They want to be able to experience these emotions when appropriate without being scared of them or blocking them, but also without allowing them to stay for longer than necessary or engaging in harmful impulsive behaviours, like self-harming, abusive behaviour, consuming alcohol or drugs.

In this context they want to reduce their mood swings and have a more balanced emotional experience. They would like this emotional balance to also be reflected in their thinking patterns, by gaining more control over their negative thoughts and reducing their black and white thinking. *“I just want to be able to… like if I'm miserable then I'm just down, I'm not wanting to die kind of thing. And then if I'm happy I'm just cheerful, not kind of flying off the walls like I've taken drugs; just to feel normal emotions”* (participant 46). They want to feel happier with their lives and be able to experience and hold on to positive emotions. *“I just want to see the sun shine, I don't want to live forever under that black cloud”* (participant 34).

#### A3. Improving relationships

Participants explained that they would like to improve their relationships, socialise more, be less isolated, build more supportive relationships in their lives, and end unsupportive or abusive relationships. Moreover, they wanted to work on their own relationship skills, develop trust towards others, be able to talk about their feelings and allow themselves to feel vulnerable in close relationships, tolerate fears of rejection and abandonment. This was perceived as particularly hard, commonly due to the lack of validating relationships in their childhood and experiences of abuse or neglect. Similarly, developing a better understanding of how their actions might impact on other people and becoming more skilful in tolerating confrontation and conflicts were also seen as signs of recovery.

*“I'm not a very mindful person, the way I was brought up, so I couldn't really take on board how other people were affected by what I was doing, or how other people are feeling. It kind of became a problem between me and my eldest son, because I kind of put a barrier up whenever he wanted to express himself, because I wouldn't understand what he's trying to say. Whereas now we are always expressing ourselves, always talking about emotions and how we feel”* (participant 7).

#### A4. Practical achievements and employment

Participants believed that having more meaningful activities in their lives is particularly important. Achieving practical things that they used to find hard, like paying their bills, managing their household, going on a holiday, using public transport and so forth made them feel more confident. *“I achieved one main goal and that was to go on holiday abroad, I went on an aeroplane! Two weeks ago I achieved a great big goal- you could think it's silly but it's big to me- I actually went on the bus for the first time after 25 years!”* (participant 1).

They also wanted to work towards finding a job and making progress in their career, as this makes them feel more competent and ‘normal’. *“I still haven't managed to get back to work and I can't see friends, I've been cut off because I've stopped working. Not having a job means being financially dependent and just affects your self-esteem, like knowing you haven't really got the confidence to go out”* (participant 12).

#### A5. Reducing suicidality, self-harming and other symptoms

Some clinical outcomes linked to BPD symptoms were also seen as important by service users. Thus improvement in a wide range of areas, including suicidality, self-harming, alcohol and drug use, eating problems and post-traumatic stress symptoms, was perceived as part of recovery. People wanted to reduce such behaviours and gain more control over urges to engage in them. *“Stopping self-harming was one of my goals… It got to the point where it was a thing as an addiction. The moment I felt even the slightest bit of stress I was cutting, so even I knew it escalated and I needed…I don't think I necessarily wanted to stop but I wanted to control it, ultimately it stopped”* (participant 10).

### B. Balancing personal goals of recovery versus service targets

Some participants thought that there was a clash between their personal aspirations and the focus of treatment. They felt that therapy did not address all problems they were struggling with. Some treatments were experienced as focusing almost exclusively on specific topics, i.e. self-harming or relationships (often as they were enacted in the group setting), leaving service users frustrated when they could not address other issues that were either equally or more important to them. Other common problems that service users felt were not sufficiently addressed were eating problems and past traumatic experiences. *“DBT helped, but it didn't answer all of my questions. It didn't help me to work things through myself, it didn't help me to achieve my goals really… I was trying to get over my divorce and also my relationship with my mum and men, and I was trying to work through it but it was all about other things, it was about self-harming, it was about mindfulness…”* (participant 11).

### C. How recovered do people feel?

Participants described various states of recovery, as illustrated below. These were perceived either as different stages in their journey to recovery or as reflecting their overall recovery status.

#### C1. No progress

Some participants believed that they have made no progress or not as much progress as they would have liked. *“I 'm not different to before I started treatment. I'm just fat… I'm just blowing up. I just seem so ugly, I actually feel it, I look at myself and I go, what the hell have I become?”* (participant 33). Sometimes they felt that they made progress in one area, but that might have led to deterioration in different areas. They therefore felt hopeless and frustrated with their situation. *“I sort of deal with one thing but then the other problem will escalate… like when I used to take cannabis, I cut down on my drink but smoke more cannabis; if I didn't have cannabis, I'd smoke more fags, so it's always something is replaced…”* (participant 26).

#### C2. Recovery fluctuating

Others described how their recovery fluctuates. They experienced going through phases when they feel better, in control, and more able to deal with their problems. However, these phases were then followed by periods when they feel defeated and less able to cope with life. Such fluctuations made interviewees feel worried about their future and uncertain about whether they should trust their own feelings of recovery. *“I can go through periods. Yesterday was relatively ok, today is ok so far. But before, consistently, I had a period where I couldn't actually leave the house and I was very dissatisfied and self-hating… So it's difficult to actually trust the times when I am feeling alright”* (participant 20).

#### C3. Able to deal with things in a better way but not (fully) recovered

Most interviewees felt that they have improved to a degree or in some areas, although not fully recovered. They believed that they were more aware of their problems, which helped them deal with them in a better way and ensure that their emotions do not escalate and get out of control. *“I think it's still there, so as to recovering…I actually don't see it as before and after, you can have a diagnosis of depression and then move through that depressive phase…it feels a bit more like who I am…but I'm aware, I try to keep on top of [borderline traits], I'm quite reflective in myself, I am feeling significantly more peaceful in myself, happier, I function better, day-to-day stuff I'm better at, I'm managing my own home, although there is still aspects that I struggle with greatly”* (participant 8).

Some saw this as a step in their journey towards further recovery, whereas others believed that this was the best outcome they could hope for. *“I don't think I have recovered. I think it's too much of a nice thought to recover from it. I'm dealing with it. I'm dealing with it in a different way”* (participant 4).

#### C4. Recovered

Five interviewees reported that they have recovered, although only one held this view consistently throughout the interview. *“I think I have recovered from BPD. Although I know I still find myself having the same panicky reactions to things, it's just that I can rationalise with myself more easily now. I can still overreact to situations, but the difference is I know I'm doing it. And I know that I can do something about it”* (participant 13). The remaining four said at times in the interview that they have recovered, whereas at different points they expressed more ambivalent views.

### D. Problems with the word ‘recovery’

Some interviewees believed that recovery might not be the right word to describe their progress. They felt that the term recovery implies a dichotomous classification of problems, suggesting that people either have problems or they are fully recovered. They believed that in BPD this is particularly inappropriate, as it might reflect and encourage black and white thinking, one of the symptoms of the disorder. They thought that full recovery in that sense is impossible, as they could not imagine not having some difficulties in dealing with their emotions and lives. Thinking that they are recovered following such a dichotomous definition was also seen as dangerous, as it could be unrealistic, indicate a lack of acceptance of their problems, and lead to not monitoring themselves and therefore relapsing. *“I think recovery is a very difficult word particularly with mental illnesses and I think you can recover, but I suppose I'm naturally worried that if I go and recover, I would be worried that I could think I'm wonderful now and then all of them fall from the rails, cause I'm not keeping a check of myself… I think mentally I'm always gonna have to keep in my mind that I have these issues, I have this problem, I have this diagnosis and that's not to say it's a bad thing… I don't want to say I've recovered because I don't want to give myself an opportunity to relapse”* (participant 9).

Other participants thought that especially in the context of BPD separating themselves from the disorder is particularly hard, as they have been experiencing emotional difficulties for as long as they remember. Therefore, BPD is not something they feel they can recover from, as that would mean that they would have to become a different person, which is not necessarily what they want. *“I can't imagine not having BPD. I don't remember a time in my life when I didn't feel this way. So recovery, cure… no, I don't think so. Learning how to deal with it, I'm very positive about… I don't think it will every go away. I'd have to have a personality transplant for that to happen… I'm not entirely sure I want to actually let go of certain aspects of me. I like the fact that I'm a compassionate human being. I don't want to be this involved with people's emotions, but I wouldn't want to not care. And that wouldn't be me. I'd much rather learn how to deal with it than have it taken out of me”* (participant 22).

More quotes on the above themes are presented in Box S1.

## Discussion

### Main findings

For service users with BPD recovery involved developing self-acceptance, self-confidence and self-esteem, gaining control over emotions, moods and thoughts, improving relationships, getting involved in activities and employment, and making progress in clinical symptoms, such as suicidality, self-harming, eating problems, drug and alcohol consumption. Some service users felt that some of their goals were not adequately addressed and that therapies had an extreme focus on specific topics, such as self-harming or relationships. Recovery was experienced as a dynamic process with various stages. Participants described how their recovery fluctuated, with periods with marked improvements followed by times when things were particularly hard to manage. This made them feel exhausted and disheartened, although it was often seen as a natural process in their recovery journey. Although most interviewees felt that they make continuous and gradual progress, full recovery was commonly seen as a distant goal. However, people felt hopeful that they can learn how to deal with their problems in more effective ways and keep on moving forward and making meaningful changes in their lives.

Interestingly, some service users did not find the word ‘recovery’ helpful in describing their experiences of personal development and progress. Some felt that the term implies a dichotomous classification of problems, a black and white way of thinking, in which recovery becomes the unobtainable goal of having no problems at all. Others thought that, especially in the context of BPD, separating themselves from the disorder is particularly hard, as they have been experiencing emotional problems for as long as they remember.

### Strengths and limitations

To our knowledge this is the first qualitative study exploring perceptions of recovery among people with BPD. The sample size was large (48). Interviewees were recruited from a range of specialist and generic mental health services and represented various levels of recovery. The research team consisted of researchers with various academic and clinical backgrounds and service users were involved in all stages of the study.

Nevertheless, participants were recruited only from services in East London and we do not know to what extent their experiences can be generalised to users of other services. Furthermore, the specialist services investigated included DBT and MBT and we do not know whether service users' views of these approaches apply to other specialist psychotherapies for BPD, such as transference-focused therapy (TFT) or schema therapy. Similarly, therapists' perspectives' of recovery and their views on how treatments address these were not explored. Moreover, although the response rate for participation in the study was high, we do not know whether the findings could be generalised to service users who were not able or willing to take part in research. Lastly, participants' expressed views might to an extent reflect the philosophies of the treatments they received.

### Findings in the context of previous literature

The perception of recovery expressed by service users with a BPD diagnosis, where recovery is seen as an open-ended journey and involves learning how to cope with problems and developing a meaningful life with the limitations of the disorder, reflects the definition of recovery within the wider recovery literature [27]–[28]. Similarly, some of the aspirations service users described in this study are in line with recovery goals among users of general mental health services with mainly Axis I disorders. Improvements in self-acceptance, relationships, activities and employment have been widely documented as reflecting service users' perceptions of recovery [28], [36].

Other goals, however, such as gaining control over difficult emotions and specific symptoms (i.e. self-harming) are more specific to the nature of BPD. Similarly, the meaning of self-acceptance and the development of self-confidence in people with BPD might be different to re-claiming identity after a diagnosis of mental illness among users of general mental health services [36]. Interviewees described how they struggle with shame and guilt, not as a result of having a diagnosis of mental illness, but because they find it hard to come to terms with who they are. This might mirror enduring problems in developing a sense of identity and self-compassion, which often reflects a lack of secure attachment relationships and a history of abuse or neglect among people with BPD [37]. In this context, improving relationships for this group might also be more complex than solely addressing social isolation, which is commonly discussed in recovery literature [36]. More specifically, it might also involve developing relationship skills, such as building trust, tolerating fears of abandonment and allowing themselves to feel vulnerable in close relationships. Therefore, findings from this study support the view that what constitutes recovery and the meaning of specific recovery goals might be different for service users with different diagnoses [30], [38].

A key question arising from our findings is to what extent treatments for BPD address service users' personal goals of recovery. This may be more realistically achievable by specialist psychotherapies than general psychiatric services. The two specialist psychotherapies received by the study participants – DBT and MBT– both directly address some of the goals identified in this study. DBT places an emphasis on reducing self-harming and on emotion regulation [15], whereas MBT focuses on understanding and developing relationship skills [17]. Both therapies implicitly facilitate self-acceptance and self-confidence by offering a theoretical explanation of factors leading to the development of BPD and by fostering processes like mindfulness and mentalisation respectively, which are intended to help people understand and accept themselves, their emotions and behaviours [15], [17]. DBT also targets more practical goals, like employment, but only if self-harming has resolved. It is therefore encouraging that both these therapies address at least some of the targets that are important to service users. At the same time, we believe that each therapy focuses on one main area, i.e. DBT on self-harming and MBT on relationships. (It should be noted that this interpretation of the main areas of focus for each treatment might not coincide with the views of those who developed these therapies). While this might be an effective way for therapy to stay focused and help service users make significant improvements at least in one domain, it might fail to take into account other important goals. Such overfocus on single problem areas has been highlighted as a limitation of some specialist psychotherapies for BPD [8], [26], which may contribute to treatment dropout and/or slow therapeutic progress [39].

Other specialist psychotherapies for BPD, including schema and transference-focused psychotherapy, claim a wider impact on personality structure, which goes beyond improvements in individual BPD symptoms, and a higher likelihood of full recovery [25]. Schema therapy believes that by working on negative self-schemas, service users with BPD develop a more positive sense of self and agency, which then lead to further improvements in functioning and overall quality of life [26]. TFT claims that service users achieve meaningful changes by developing their self-control and healthier representations of self and others, through working on the therapeutic relationship [22]. Whether service users agree with such claims was not assessed in the present study and needs to be explored in future research. *Conclusions*

This study identified recovery goals that service users find important. Treatments focusing on these targets may increase users' motivation and engagement with services and facilitate recovery. The findings may also guide research in this area and ensure that outcomes that are relevant to service users are evaluated and processes of achieving such outcomes are explored.

The specialist therapies for BPD that were investigated in this study (DBT and MBT) explicitly address some of the recovery goals that are important to service users. However, other goals might only be indirectly or poorly addressed. It might be unrealistic for specialist treatments to offer solutions to all problems, as these treatments might naturally have to focus on some of the most important areas to be effective. Similarly, the fact that service users did not feel that these treatments addressed all their goals might also reflect an overall slow pace of recovery in BPD and the need for long-term and comprehensive care.

In this context, service users' specific priorities might determine which specialist service might be more appropriate: i.e. for those whose main aim is to stop self-harming DBT might be more relevant, whereas those who want to work on relationships might find MBT more appropriate. Other treatment models, like schema and TFT, could also be explored based on service users' preferences. Future research should focus on understanding specific processes of change within each therapy, which might also guide treatment choice by service users [24].

Professionals might also need to work with service users towards devising comprehensive individualised case formulations, including all treatment targets that are important to them, their priorities and long-term plans on how their targets might be met and which services might be involved. Specialist services for BPD might be a starting point, but other services, including eating disorders, drug and alcohol, trauma services, or employment schemes might also be involved at different stages of recovery.

## Supporting Information

Box S1 **Service users' perspectives of recovery in Borderline Personality Disorder (BPD).**
(DOC)
