# Supplementary material for: Recovery in Borderline Personality Disorder (BPD): A Qualitative Study of Service Users' Perspectives
Source: PLoS One. 2012 May 17;7(5):e36517. doi: 10.1371/journal.pone.0036517 (PMC3355153; doi:10.1371/journal.pone.0036517)
Supplement: Box S1 — Service users' perspectives of recovery in Borderline Personality Disorder (BPD). (DOC) [file pone.0036517.s001.doc]

# Box S1. Service users’ perspectives of recovery in Borderline Personality Disorder (BPD)

| A. Personal goals and/or achievements during recovery |
| --- |
| A1. Accepting self and building self-confidence |
| *“Treatment helped me understand the traits of borderline and understand that they were part of a psychiatric diagnosis that lots of other people find, it’s not just me. By understanding them, I was in a better place to be able to deal with the world… I’m now better at understanding when I do things and what are the things I am doing that are borderline traits”* (participant 3).  *“I’ve started mellowing down a bit more and be more “this is your life, ignore all the sarcastic remarks that go on in your head, you are who you are, and then basically just to be happy with your life rather than miserable all the time”. I’m not as miserable now as I used to be everyday. Now it’s a lot better, I can laugh, I can joke, whereas before I didn’t see anything fun there, I used to think the world was on top of me, I owe the world a favour, silly things like that… I don’t feel as guilty as I used to -sometimes I do but not to the extent where I’d want to rip myself, I’d want to like kill myself and just end it all or hurt myself, punish myself…”* (participant 42). |
| A2. Taking control of emotions, mood and negative thinking |
| *“Sometimes when I get an emotion I don’t know why I’m feeling that emotion, I can cry but its not something I ve seen that’s sad or I haven’t just lost someone, none’s just passed away or none’s particularly hurt me or beat me up. I’d like to know the reason why them emotions come in, I think that’s what I’ve never been taught and then if I get angry it’s like, I can’t remove myself from that situation, I wish I could just think right, I gotta get out of here, go for a walk, but that doesn’t come in because by the time I’ve felt that, my anger’s so up there! I d just like to know that, ooh that’s coming now, I know that feeling, right lets do something now, change it!”* (participant 26)  *“I think one of the really good things that has come out of this is… a lot of things for me were black and white in the sense that everyone was either happy or should be very happy or you’re really miserable, and I sort of met in the middle really and thought you know ‘you can’t be happy, gloriously happy every day of the week, every hour of the day and you shouldn’t be miserable either’, so I am happy, I’m more satisfied with my life, there’s good days and bad days as with everybody but I recognise it’s not a drama, it’s just a bad day and I get on with it, then sort of move on”* (participant 9). |
| A3. Improving relationships |
| *“Things between me and my family were quite bad when I came here, I wasn’t able to see my niece for months because I was acting to be crazy, hardly speaking to my sister… but I see them once a week and we’re really close now and that wouldn’t have happened before”* (participant 6).  *“I suppose recovery is if… I can trust people in our relationship, mentalise better, think before I open my mouth or judge a person, not everyone’s the same. And what I can gain is that I can trust the relationship, trust my kids more, the people who care for me, and just realising that… always know the people who care, ’cause I have these doubts, like nobody wants me, and nobody cares”* (participant 21). |
| A4. Practical achievements and employment |
| *“In the past I didn’t want to see people. I didn’t want to go out. I stayed in the house for so many hours, in the bedroom, and I didn’t want to see nobody. But now I told my husband, I’m going out, I don’t want to stay in the house, ‘cause I feel bad. I started to go out alone for something, to go to the shop near the house. But sometimes I need support from my husband to go out, I don’t feel confident”* (participant 39).  *“I hope I can get off my medication one day and get a job. I want to be a normal person. I’d like to work for mental health, to help other people”* (participant 28) |
| A5. Reducing suicidality, self-harming and other symptoms |
| *“My goal was to reduce self-harming because I hated it. I hated it, because I scar. For a bit I dressed to sort of cover it. Even today I’ve got a scarf on, because I’ve still got sort of scars on my neck from where I’ve constantly been sort of scratching at my skin. So, yeah, I don’t like it, um… yeah, it was definitely a goal. I mean I spent hours doing it, literally hours, and be, sort of, almost red raw from scratching at my skin”* (participant 25).  *“I had loads of goals, one of them was to reduce drinking, ’cause I was drinking overly too, too much every single day … I mean obviously I was there drinking everyday, I’d given up basically, I was giving up about life and then I thought to myself, I thought, okay I can give treatment a chance”* (participant 23). |
| B. Balancing personal goals of recovery versus service targets |
| *“A lot of being here is focused on relationships and I do agree that it’s a big part of it, but there are other aspects as well like low mood, self-esteem, confidence, work, and they need to be worked on as well… sometimes a lot of the focus can be on the actual running of the community, which I know is important but sometimes you need to work on the other aspects as well…some issues are not taken on board, like with the eating, I mentioned it many times and I find it very difficult to talk about, and I wouldn’t eat with them, so it was obvious I was struggling. And it would be raised in the sense “we’ve noticed you didn’t eat”, but there was nothing like practical like a dietician or getting to the bottom of it. So sometimes I think things are raised but they are not dealt with, so then you’re left and you think “Is there a point of bringing it up? Or what’s the point, I have to deal with it by myself”* (participant 14).  *“It was identified that I had some post traumatic stress disorder and it was very clearly told to me “we can’t deal with this, you are here for borderline, we can’t really talk about the domestic violence, we can’t talk about what went on between the two of you and what you’ve suffered, we can’t deal with the post traumatic stress…so that’s kind of like out of the window, we can only deal with the borderline” and that was very difficult for me for a long time, because I’m actually feeling I have all the symptoms of post traumatic stress and all of the problems of the domestic violence but there was no area within DBT to discuss that”* (participant 8) |
| C. How recovered do people feel? |
| C1. No progress |
| *“I've got worse, not better. In every way, I think! Definitely worse mentally, definitely. I just know I'm getting worse, not better”* (participant 34).  *“No, I haven’t recovered. I hate the person I am at the moment, I don’t like who I am. I mean I used to be the person that would cheer you up, could be high in her soul and everything and I’ve gone from that to not being able to deal with a lot of things. I can’t stand it”* (participant 43). |
| C2. Recovery fluctuating |
| *“I think that I got through a period where I am well in terms of I can function normally and I become very positive about my life and make plans and be sociable and then I think because I can’t deal with the outside world, I really can’t, it just gets very difficult and whatever progress I’ve made something happens and I just go back... And it becomes exhausting and now I’m exhausted”* (participant 38).  *“I don’t definitely feel better, I just feel a little bit better in myself. I won’t say it’s because of this treatment that I’m feeling great, I mean like I said, I go up and down, so this week I could be up, I’ve got no problems... and then next week I could be as down as a Cheshire cat”* (participant 40). |
| C3. Able to deal with things in a better way but not (fully) recovered |
| *“From past experiences I know that my problems aren’t going to go away, it’s not getting rid of the problems, it’s being able to cope with them. Seeing problems in a more positive light, or how to deal with different problems. So I don’t think that coming here is going to cure me of violent depression or personality disorder, it’s always going to be there, but it’s about seeing a better outlook on it”* (participant 18).  *“Treatment certainly has helped me to become much more aware of tendencies and ways of thinking and given me sort of responses that I can go to when I recognise certain ways of thinking or acting. So it’s an improvement of degree, because it’s a lot of these things that, although maybe I know the technique, say there is something I find particularly that brings up a lot of shame, I spend a lot of time in avoidance. So there is a lot of improvement nearly done, I mean still I do think I’ve improved in quite a few ways, things like less social isolation but again…there is a lot to still watch out for”* (participant 2).  *“I know I had a problem in the past with dyslexia, my mum is not talking to my dad, my dad is not talking to my mum… and all those things haunt you, there is something wrong with you, and you carry all this sort of baggage with you, and it needs sorting. You won’t get rid of it, you won’t lose it or anything like that, but you can sort it, you can handle it better, and that is how DBT tidies you up, it sorts you out … you can understand why you did this, you can understand why people did that to you, and it is through understanding that you can declutter sort of thing. Don’t ruminate on the past, you can’t take it with you all the time, and I think that I have learned through DBT that I can do that”* (participant 27). |
| C4. Recovered |
| *“Sometimes I feel like I have recovered, I don’t meet the criteria [for BPD], sometimes I feel it… I feel I can interact with people, in a way it is to me it feels like a problem, but people that meet me don’t actually think there’s anything wrong, that I’m confident, I’m able and capable, so I can actually cope in situations. So sometimes I feel like I do have issues relating to people and it does need work on, but sometimes I feel like I haven’t felt depressed for a long time, I’ve not been on antidepressants for a year and I feel I’m coping with this, so there is a level of me that doesn’t feel I meet the criteria and when I compare myself to how other people in the group interact in groups, I feel like I’m maybe a step above them in that service”* (participant 14)  *“I think I have recovered. I would say that I have, yeah. It’s me probably not feeling as down anymore, or depressed, me I’ve stopped harming myself… but that’s only because I think that I knew I was pregnant, and now I’ve got my daughter, which actually makes me look at bigger things and think that it’s not only me that I have to think about and she helps me kind of a lot, even though she’s not doing much, but at the moment it’s like she keeps me occupied”* (participant 24). |
| D. Problems with the word recovery |
| *“I’ve certainly made a vast improvement but I don’t know that recovery would be the right word. I certainly suffer from depression, and have done for a number of years; even if that was to completely go, because I was aware of the way I have felt, the way I have acted and the things that I’ve done, they’re memories that I have and they are going to stick with me forever, just because of the nature of memories. I see the same with personality disorder. The way I acted and the way I treated people and interacted with people in different environments has certainly changed and improved an awful lot and even my friends say that I’m much more stable and level person but whether it’s a full recovery or not, I don’t know”* (participant 5).  *“[When you asked if I feel recovered] it was difficult to answer yes or no, because... I don’t know, like there are improvements, say self-harm, but then there are other things that might be still included in borderline that I can’t even imagine not having... So that’s actually playing into my mind sort of like the black and white thinking thing. And I know recovery is what I want, but it seems like teasing in a way”* (participant 20). |
